# Circadian course of the P300 ERP in patients with amyotrophic lateral sclerosis - implications for brain-computer interfaces (BCI)

**DOI:** 10.1186/s12883-016-0782-1

**Published:** 2017-01-07

**Authors:** Helena Erlbeck, Ursula Mochty, Andrea Kübler, Ruben G. L. Real

**Affiliations:** 1 Institute of Psychology, University of Würzburg, Würzburg, Germany; 2 Institute of Medical Psychology, University of Tübingen, Tübingen, Germany; 3 Institute of Medical Psychology and Medical Sociology, University Medical Center Göttingen, Waldweg 37, 37073 Göttingen, Germany

**Keywords:** Amyotrophic Lateral Sclerosis, ALS, P300, Auditory, Visual, Brain computer interface, BCI

## Abstract

**Background:**

Accidents or neurodegenerative diseases like amyotrophic lateral sclerosis (ALS) can lead to progressing, extensive, and complete paralysis leaving patients aware but unable to communicate (locked-in state). Brain-computer interfaces (BCI) based on electroencephalography represent an important approach to establish communication with these patients. The most common BCI for communication rely on the P300, a positive deflection arising in response to rare events. To foster broader application of BCIs for restoring lost function, also for end-users with impaired vision, we explored whether there were specific time windows during the day in which a P300 driven BCI should be preferably applied.

**Methods:**

The present study investigated the influence of time of the day and modality (visual vs. auditory) on P300 amplitude and latency. A sample of 14 patients (end-users) with ALS and 14 healthy age matched volunteers participated in the study and P300 event-related potentials (ERP) were recorded at four different times (10, 12 am, 2, & 4 pm) during the day.

**Results:**

Results indicated no differences in P300 amplitudes or latencies between groups (ALS patients v. healthy participants) or time of measurement. In the auditory condition, latencies were shorter and amplitudes smaller as compared to the visual condition.

**Conclusion:**

Our findings suggest applicability of EEG/BCI sessions in patients with ALS throughout normal waking hours. Future studies using actual BCI systems are needed to generalize these findings with regard to BCI effectiveness/efficiency and other times of day.

## Background

For people with advanced neurodegenerative diseases or in states of severely reduced mobility due to accidents or illness, a brain-computer-interface (BCI) might be the only means to communicate with their environment. A BCI uses brain signals most often recorded with electroencephalography (EEG) and converts them into control signals replacing, restoring, or enhancing the natural output of the central nervous system the patient (end-user of BCI technology) is no longer able to perform [1] (see also the BNCI H2020 roadmap). For example, the so-called P300-speller, a BCI application to allow the spelling of words on a computer screen, is one of the most commonly used devices and has been applied in healthy participants as well as in patients [2–6]. It relies on a positive deflection about 300 ms after a rare stimulus in a visual oddball paradigm comprising irrelevant frequent and relevant rare (target) stimuli (P300 ERP, [7, 8]).

In addition to the visually based P300 speller, there have also been efforts to design a BCI solely relying on auditory stimuli for users with impaired vision or eye gaze control [9–15]. Until recently, auditory BCIs faced the problem of a limited amount of classes, i.e. differentiable choices they present to the end-user of BCI [16]. However, Höhne and Tangermann introduced the CharStreamer system, which is based on 30 spoken sounds of letters and actions and demonstrated a competitive spelling speed. Halder and colleagues [17] showed that even patients with severe motor impairment including those with amyotrophic lateral sclerosis (ALS) could control an auditory speller with high accuracy provided several training sessions.

Patients with neurodegenerative diseases, specifically those diagnosed with ALS are a major target group for BCIs. ALS affects neurons of the motor system and beyond, leading to progressing muscle weakness and atrophy [18]. In its late stages, patients are left unable to move or breathe while being fully aware of themselves and the environment. Thus, for these patients a BCI solely relying on brain activity may be the only way to communicate their thoughts and wishes.

In general, BCI performance is not only influenced by soft- and hardware factors, e.g. choice of equipment and classifiers, but also by physiological and psychological variables related to the generation of the EEG input signal [19]. For example, the frequently used P300 ERP depends on attention and working memory processes [20, 21], with reduced attention / high working memory load being associated with lower amplitudes and prolonged latencies [22]. In addition to motor impairment, patients with ALS may also experience respiratory dysfunction, disrupted sleep, and fatigue [23], which in turn may limit attentional and working memory performance [24]. However, it is unclear how relevant these findings are in an applied setting; some studies reporting P300 amplitudes comparable between healthy participants and ALS patients, and others showing reduced P300 amplitudes in patients with ALS [25–27]. Similarly, some studies found prolonged latencies in ALS [27, 28], while others revealed no such difference [29, 30].

Given the objective of BCIs to become a viable means for long-term home based communication in patients with ALS, a second potentially important factor concerns the sensitivity of attention and working memory to circadian variation. Whereas behavioral measures of these processes generally indicate declining performance throughout the day [31], it is unclear whether these changes are also associated with reduced amplitudes/prolonged latencies in the P300 ERP. Some studies of healthy participants suggest sensitivity of the P300 to time of day, whereas other studies found no such an effect [32–35]. In addition, and despite its potential importance, no information on the sensitivity of the P300 to time of day is available from patients with ALS.

Finally, the factor of modality may become relevant when evaluating the suitability of a BCI for ALS patients. For example, there is evidence that –in healthy participants-, the P300 is influenced by modality, with smaller amplitudes and shorter latencies in the auditory domain [36] (but see [37]). Although vision is usually unaffected by ALS, some late stage patients are easily exhausted when using vision based communication devices. For these patients, reliance on the auditory modality may be one of the few alternatives left [38]. Thus, a systematic comparison of the P300 ERP between modalities and time of day is warranted, as this information may be important for researchers when scheduling EEG/BCI sessions and other psychophysiological experiments.

To provide such data, we recorded visual and auditory P300 in patients diagnosed with ALS and age matched healthy participants at four different time points within 1 day in their homes. Specifically, we hypothesized that

1. there is a decline in P300 amplitudes and an increase in P300 latencies over time (main effect of time of the day).
2. the effect of the daytime is stronger in ALS patients than in healthy controls (group x time interaction).

Data presented in this paper were taken from Ursula Mochty’s dissertation [39] and reanalyzed.

## Methods

### Participants

A sample of 14 ALS patients and 14 age and sex matched healthy control subjects participated in the study. We only included ALS patients who were still able to communicate a “Yes” or “No” answer in any modality, thus, no ALS patients in the complete locked-in state were included. All participants had normal or corrected to normal vision. ALS patients were between 40 and 73 years old (*M* =67.93, *SD* = 10.46), healthy participants were between 38 and 79 years of age (*M* = 58.07, *SD* = 10.69; *t* = −0.034, *DF* = 26, *p* = .972). All ALS patients were assessed with the revised ALS functional rating scale (ALSFRS-R; [40]). Scores varied between 2 and 36 (*M* = 21.79, *SD* = 10.53), with lower scores indicating more severe physical impairment.

### Procedure and stimuli

The study was conducted at the participants’ home. Participants sat in a comfortable chair with a computer screen in front of them. The experiment consisted of four identical sessions conducted at 10, 12 am, 2 and 4 pm. Each session lasted 12 min and started with the recording of 4 min of resting EEG, followed by EEG recording during a visual (4 min) and auditory oddball (4 min). During oddball paradigms, participants were instructed to silently count the deviant stimuli.

#### Auditory oddball

Auditory stimuli were 160 standard (1000 Hz) and 40 deviant (2000 Hz) tones (rectangular pulse of 50 ms duration) presented in pseudo-random order with the restriction that any two deviant tones were separated by at least one standard tone. Stimulus onset asynchrony (SOA) was 1200 ms with a random latency jitter of ± 0.15 s to avoid habituation to a certain SOA [41].

#### Visual oddball

Visual stimuli were short (50 ms) presentations of the letters “H” (40 times, deviant) and “S” (160 times, standard) in white font (120 pt) on a black background in the centre of a computer monitor [42]. Stimulus onset asynchrony (SOA) was 1.2 ± 0.15 s (see above).

### Data acquisition

EEG was recorded using a g-tec USB amplifier (g-tec, Schiedlberg, Austria) with a sampling rate of 256 Hz (band-pass filter: 0.01 and 30 Hz) from 16 electrodes placed at Fp1, Fp2, F3, Fz, F4, T7, C3, Cz, C4, T8, CP3, CP4, P3, Pz, P4 and Oz referenced to the right-earlobe (ground at left mastoid). Impedances were kept below 5 kΩ.

### Data processing and statistical analysis

**Fig. 1:**
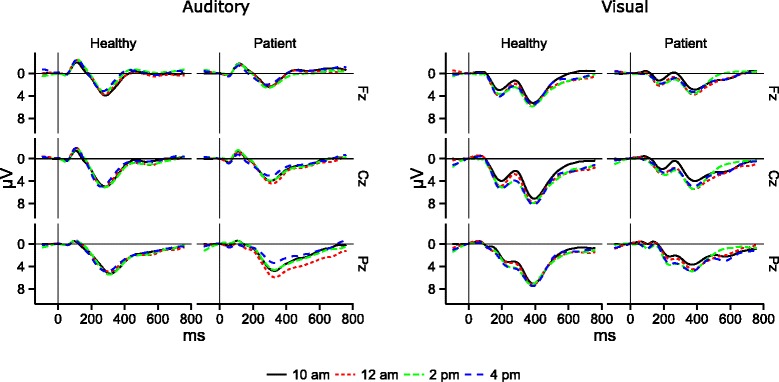
P300 difference curves (deviant-frequent), for patients with ALS and healthy participants

Offline, EEG was epoched into 900 ms long intervals beginning from −100 ms before the start of a stimulus, corrected for eye movements [43], and aligned to the 100 ms long pre-stimulus baseline. Trials with voltages exceeding ± 60 μV were rejected as artifacts, remaining trials averaged per condition, and the difference obtained (deviant – standard, see Fig. 1). This difference between the curves elicited by the standard versus the deviant represents the decisive factor for BCI classifiers to differentiate responses by the participants. In the auditory condition, peak positive amplitudes and latencies during the analysis interval ranging from 290 to 350 ms post stimulus onset were detected and exported for statistical analysis. For the visual condition, the interval lasted from 300 to 600 ms post stimulus onset. EEG data analysis was performed with Brain Vision Analyzer (Brain Products GmbH, Gilching, Germany).

Amplitudes and latencies were analyzed at midline electrodes (Fz, Cz, Pz) using repeated measures ANOVAs with within-subject factors time (10, 12 am, 2, 4 pm), region (frontal, central, parietal), modality (auditory, visual) and the between-subjects factor group (patients with ALS, healthy participants), and we report Greenhouse-Geisser—corrected p-values (after Mauchly’s Test). Effect sizes are reported as eta-square (η^2^, [44]), and Cohen’s d [45]. Marginal means [46] with degrees of freedom based on the underlying ANOVA model were used for follow-up analysis and p-values of post hoc tests were Tukey corrected for multiple comparisons. Inspection of amplitudes (Tukey boxplots, k = 1.5, [47]) indicated extremely high values for two participants in the patient sample, which were therefore excluded from all amplitude and latency analyses. Similarly, inspection of the distribution of latencies indicated potentially outlying values for five patients with ALS and five healthy participants. Since we deemed it unfeasible to exclude such high a number of participants, the results of the repeated measures ANOVA on latencies was replicated using a permutation based repeated measures ANOVA-equivalent, which confirmed the results of the parametric ANOVA.

The correlation between ALSFRS-R and P300 mean amplitude/latency was based on the respective averaged Fisher Z-transformed Spearman correlation coefficients and evaluated using a t-test. EEG curves in Fig. 1 were 10 Hz butterworth low-pass filtered for visual presentation. Statistical analyses were performed in R [48] using the ez [49], lsmeans [50], lmPerm [51], and signal packages [52].

## Results

### P300 amplitude

**Fig. 2:**
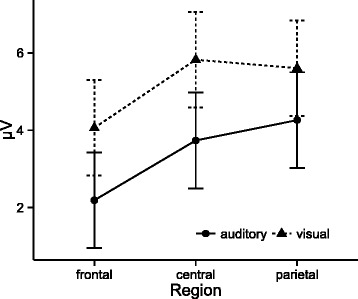
P300 amplitude across modalities and regions. Error bars represent 95% confidence intervals

Analysis of P300 amplitudes indicated significant main effects of modality (*F*(1,24) = 7.23, *p* = .013, η^2^ = .07) and region (*F*(2,48) = 17.62, *p* < .001, η2 = .06 see Fig. 2), and a marginally significant interaction of group, time, modality, and region (*F*(6144) = 2.17, *p* = .049, η2 = .001). Follow-up analysis indicated that amplitudes in the auditory modality were on average smaller than in the visual modality (*t* = −2.69, *DF* = 24, *p* = .013, d = −.22), and amplitudes at Fz were smaller than at Cz (*t* = −4.90, *DF* = 48, *p* < .001, d = −0.48) and Pz (*t* = −5.35, *DF* = 48, *p* < .001, d = −0.53), but comparable between Cz and Pz (*t* = −0.45, *DF* = 48, *p* = .894, d = −0.04). Follow-up analysis (per modality and region) on the interaction of group, time, modality, and region did not reveal significant differences in amplitudes between time of day and group (all *p* > .20).

### P300 latency

**Fig. 3:**
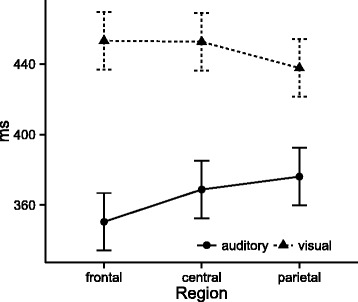
P300 latencies. Error bars represent 95% confidence intervals

Analysis of P300 latencies revealed a significant main effect of modality (*F*(1,24) = 119.13, *p* < .001, η^2^ = 0.38), an interaction of modality and region (*F*(2,48) = 6.07, *p* = .011, η^2^ = 0.02, see Fig. 3), but no effects of group, time, or any of their interactions. Follow-up analysis indicated that latencies in the auditory modality were always shorter than in the visual modality at all electrodes (all *p* < .001), but the latency difference between the two modalities was larger at Fz than at Pz (*t* = 3.48, *DF* = 48, *p* = .003, d = 0.68) with no significant differences between either Fz and Cz and Cz and Pz (all *p* > .156).

### Relation to physical impairment (ALSFRS-R)

To test for potential interrelations between severity of ALS (ALSFRS-R score) and P300 amplitude/latency, we calculated correlational analyses in the patient sample. Disease severity did not correlate with P300 peak amplitude (*r* = .21, *t* = 0.68, *DF* = 10, *p* = .745) or latency (*r* = −0.09, *t* = −0.29, *DF* = 10, *p* = .389).

## Discussion

The present study was conducted to investigate whether time of the day and modality (visual vs. auditory) should be considered as factors influencing P300 amplitude and latency when conducting BCI sessions in patients with ALS.

Our hypotheses predicted a general decline of P300 amplitudes and increased latencies throughout the day, and that this proposed effect would be even more pronounced in patients with ALS. Our analyses neither revealed significant differences in ERP amplitudes/latencies between patients with ALS and healthy participants, nor changes with time of day. Instead, we only found that in the auditory domain, amplitudes were smaller and latencies shorter than in the visual domain. P300 amplitudes in both modalities were more positive at Cz and Pz than at Fz.

These results indicate that in patients with ALS P300 amplitudes comparable to those of healthy participants can be elicited in both modalities, which supports the feasibility of a P300 BCI also in late stage ALS. Although some previous studies obtained similar results [28, 30], this result it still surprising because the majority of studies reports diminished amplitudes and an impaired capacity of sustained attention and working memory in patients with ALS [25–27, 29]. One possible reason for these comparable amplitudes between ALS patients and healthy participants might be the home environment in which measurements were undertaken. It might be possible that a familiar environment enables the patients to better concentrate on the stimuli and tasks as compared to a laboratory setting.

In a previous study, Wesensten and colleagues [34] found decreases in auditory P300 over the course of the day. However, these findings were challenged by Geisler and Polich [35] who concluded from their study, that auditory P300 amplitudes did not depend on the mere time of day, but on pulse-rate, or body temperature and food intake, both often correlating with time of the day. Food intake, heart rate, or body temperature were not assessed in our study, so no quantification of the effect of these factors can be presented. However, judging from the absence of an effect of time of day, it would appear that their combined effect would be too small to warrant differential scheduling of BCI by time of day. In sum, P300s seem to be elicited irrespective of the time of the day when presented to participants in their home environment.

Although auditory stimulation led to overall smaller P300 amplitudes, amplitudes in ALS patients were still comparable to those of healthy controls. Previous studies have already stressed the need to establish auditory BCIs in ALS patients [53, 54], as in late stages of ALS, patients may also loose reliable control over eye movements. As a consequence, in ALS patients as well as in other behaviorally non-responsive patients, the auditory channel is usually better preserved [55] or might even be the only remaining channel to establish communication [56]. Application of auditory BCIs of different kinds have shown promising results in healthy participants [9, 15, 57–59] and auditory P300 BCIs based on the tone segregation phenomenon [60] or animal sounds were also brought to patients [59].

### Limitations

Data collection took place at the participants’ homes, and analyses were deliberately restricted to midline positions. This was done bearing in mind real-world application scenarios for BCIs, especially with regard to patient’s desire for limiting the number of electrodes as much as possible [61]. While similar restricted setups have been shown to be sufficient for achieving BCI control in patients with ALS [62], our results are still only tentative given that classification performance was not evaluated. In addition, we highlight that while time of day is certainly a convenient indicator of patients’ fatigue, other, more direct measures could have been used. For example, analyses involving “hours awake”, self-reported tiredness, or even physiological indicators of circadian variation [34] as covariates might yield different results, and, hence, such measures might be included in future research. Finally, the low number of patients might limit generalization of results.

## Conclusion

Taken together, the data presented in this study suggest that P300-based BCIs could be applicable in patients with ALS throughout normal waking hours (10 am–4 pm). Patients presented with P300 amplitudes and latencies comparable to those of healthy participants in both, the auditory and the visual domain. Furthermore, there was no correlation between the constraints in physical functioning measured by the ALSFRS-R and P300 physiology. This means that reliable P300 potentials can be elicited also in late stages of the disease, thus preserving the basic requirements for BCI use. This is also supported by single-case studies which evaluated the usability of BCIs during long-term independent home use [63, 64].

In sum, the present results show the feasibility of P300 ERP recordings in patients with ALS throughout the day, and, hence, the P300’s potential usefulness as a BCI input signal in long term independent home use scenarios.

## Abbreviation

ALS: Amyotrophic lateral sclerosis
ALSFRS-R: Revised Amyotrophic Lateral Sclerosis Functional Rating Scale
BCI: Brain computer interface
EEG: Electroencephalography
ERP: Event related potential
SOA: Stimulus onset asynchrony
